# SIRI and SII as potential biomarkers of disease activity and lupus nephritis in systemic lupus erythematosus

**DOI:** 10.3389/fimmu.2025.1530534

**Published:** 2025-01-31

**Authors:** Chi-Hui Yang, Xin-Yi Wang, Yi-Hui Zhang, Ning Ding

**Affiliations:** Department of Laboratory Medicine, Ruijin Hospital, Shanghai Jiao Tong University School of Medicine, Shanghai, China

**Keywords:** disease activity, inflammatory biomarkers, lupus nephritis, predictive markers, systemic immune-inflammation index, systemic inflammation response index

## Abstract

**Objectives:**

Inflammation is important in the development of systemic lupus erythematosus (SLE). Systemic inflammation response index (SIRI) and systemic immune-inflammation index (SII) are novel clinical markers of inflammation with prognostic value in different diseases. However, the value of SIRI and SII as inflammation predictors in SLE remains unclear. This study explores the SIRI and SII as potential biomarkers for SLE.

**Methods:**

Data from 280 individuals, including newly diagnosed SLE patients and healthy controls, were collected and divided into three groups: SLE without lupus nephritis (NLN) group (n=93), lupus nephritis (LN) group (n=96) and healthy control group (n=91). Differences in SIRI and SII among the three groups were compared. Logistic regression and Pearson linear analysis were used to analyze the predictive value and correlation of SIRI and SII with SLE and systemic lupus erythematosus disease activity index 2000 (SLEDAI-2K). Receiver operating characteristic (ROC) curves evaluated SIRI and SII in predicting SLE, SLE disease activity, and LN.

**Results:**

The SIRI and SII values were significantly higher in the LN group compared to the NLN group (p<0.01). SII had the largest area under the ROC curve for predicting LN (AUC: 0.6775, 95%CI: 0.6020 - 0.7531). Logistic regression analysis showed SIRI and SII as independent risk factors for LN. Pearson linear analysis indicated SIRI and SII were positively correlated with SLEDAI-2K (r_SIRI_=0.25, r_SII_=0.24, p<0.05).

**Conclusions:**

SIRI and SII are biomarkers of disease activity and renal involvement in SLE patients that can be used to evaluate and predict for SLE occurrence, disease activity, and lupus nephritis occurrence assessment.

## 1 Introduction

Systemic lupus erythematosus (SLE) is a chronic autoimmune disease characterized by the accumulation of autoantibodies. It often manifests with the involvement of multiple systems and organs (1). Untreated or improperly managed SLE can cause irreversible, life-threatening organ damage. Lupus nephritis (LN), a chronic kidney disease, is one of the most common and serious organ complications of SLE, affecting about 60% of patients (2, 3). Main clinical manifestations include proteinuria, gross/microscopic hematuria, casts in the urine, impaired renal function. The main challenge in early SLE diagnosis is that delays can result in missed opportunities for effective treatment. Renal biopsy is a commonly used and reliable technique for diagnosing LN. However, this invasive procedure itself causes certain trauma to patients, making the timing of its use an urgent issue to solve clinically. Several international standards are established for assessing SLE disease activity, including the systemic lupus erythematosus disease activity index (SLEDAI-2K), systemic lupus activity measure (SLAM), and the British Isles lupus assessment group scale (BILAG), with SLEDAI-2K being the most widely used. However, clinical laboratory indicators such as urine protein levels, anti-dsDNA antibodies, complement level, creatinine (CREA), and blood urea nitrogen (BUN) have limitations and are relatively low in sensitivity and specificity, and cannot accurately distinguish between active and chronic kidney injury promptly (4, 5). Studies have shown that certain inflammatory factors and chemokines, such as TWEAK, MCP-1, NGAL, OPG, Lipocalin-2, IP-10, and CXCL-16, are involved in the pathogenesis of lupus nephritis. These factors could potentially be biomarkers for lupus nephritis, but their expression levels are influenced by various factors, including genetics, environment, and immune status (6). Therefore, novel non-invasive biomarkers are urgently needed to predict disease activity and the onset of nephritis. Ideally, these biomarkers will demonstrate significant and specific changes in the early stages of the disease, and thus evaluating the progression.

Systemic inflammation response index (SIRI) is a highly sensitive marker of inflammation across various diseases, calculated from monocytes and neutrophil-lymphocyte ratio including cancer, cardiovascular disease, and infection (7, 8). Systemic immune-inflammation index (SII), derived from platelet counts and neutrophil-lymphocyte ratio, along with SIRI, provides a more comprehensive representation of the immune-inflammatory condition. In addition, neutrophils, lymphocytes, and platelets are important components of the inflammatory response, which contributes to the progression of many autoimmune diseases (9, 10). Expressing these hematological indicators as ratios or indices enhances their predictive value (9, 11). SII and SIRI combine multiple blood routine parameters, enabling a more comprehensive assessment of the immune-inflammatory status and facilitating a more accurate evaluation of SLE disease activity. In contrast, C-reactive protein (CRP) and erythrocyte sedimentation rate (ESR) are non-specific inflammatory markers, and their levels can be influenced by various factors such as age, gender, and physiological state, which may lead to deviations in assessing SLE disease activity.

SII and SIRI are derived from various ratios and counts of immune cells, including neutrophils, lymphocytes, and platelets. These cells are essential for immune responses and often change in autoimmune diseases. Neutrophils typically increase during inflammation and infection, contribute to tissue damage by releasing reactive oxygen species and proteolytic enzymes. In SLE, neutrophils can form neutrophil extracellular traps (NETs), worsening inflammation and tissue injury (12). Lymphocytes, particularly T cells and B cells, are important to adaptive immune response. Dysregulated lymphocyte function and proliferation can lead to the production of autoantibody and sustained inflammation. The counts and ratios of lymphocytes may indicate the level of immune activation and autoimmunity in SLE patients (13). Monocytes, which differentiate into macrophages and dendritic cells in tissues, are essential for phagocytosis and cytokine production. Monocyte levels elevated in inflammatory conditions like SLE, contributing to tissue damage and chronic inflammation (14). Platelets are involved in inflammation and hemostasis. Their activation can cause vascular damage and intensify inflammatory responses. In SLE, increased platelet counts or activation may be associated with disease activity and organ involvement (15).

In recent years, the use of inflammatory biomarkers for the identification and prognosis of immune system diseases has become a hot research topic. However, the association between SIRI and SII with SLE disease activity and LN has been rarely studied. This study aims to investigate the relationship between SIRI and SII with disease activity and LN in SLE patients. By assessing these indicators, we seek to evaluate the disease progression of SLE and provide a solid basis for deciding the need for invasive testing.

## 2 Materials and methods

### 2.1 Study participants

From January to December 2023, 189 patients with newly diagnosed and treated systemic lupus erythematosus (SLE) at Ruijin Hospital, affiliated with Shanghai Jiao Tong University were enrolled. A retrospective analysis was conducted. All enrolled patients met the classification criteria for SLE by 2019 European League Against Rheumatism/American College of Rheumatology (EULAR/ACR) Classification Criteria for Systemic Lupus Erythematosus (SLE). They were divided into the lupus nephritis group (LN group) and the non-lupus nephritis group (NLN group) based on the presence of lupus nephritis. The diagnostic criteria for LN included: meeting the 2019 EULAR/ACR SLE classification criteria; persistent proteinuria > 0.5 g/d or urine protein > 3+ in routine urinalysis; and/or cellular casts, such as red blood cell casts, granular casts, or mixed casts; and/or renal biopsy pathology confirming LN. Exclusion criteria were: (a) presence of other autoimmune diseases such as systemic sclerosis, rheumatoid arthritis, primary Sjögren's syndrome, myasthenia gravis, or mixed connective tissue disease; (b) presence of end-stage renal disease, severe liver disease, severe cardiovascular or cerebrovascular disease; (c) presence of malignant tumors; (d) repeated use of antibiotics within the past month; (e) presence of thrombosis within the past month; (f) previous use of glucocorticoids or immunosuppressants; (g) incomplete clinical data; (h) psoriatic arthritis; (i) other causes of kidney affection and proteinuria (such as diabetic nephropathy, nephrotic syndrome, multiple myeloma and myloidosis). Additionally, 91 healthy individuals undergoing physical examinations during the same period were included as a control group. This study complied with the Declaration of Helsinki and was approved by the Ethics Committee of Ruijin Hospital [Ethics Number: (2019) (54)], with informed consent obtained from all participants.

### 2.2 Data collection

#### 2.2.1 Sample collection

Venous blood samples were collected from each participant in the early morning after fasting. Each sample consisted of 9 ml, with 5 ml allocated to serum separation and procoagulant tubes (SST) and 2 ml each to EDTA anticoagulant tubes. All samples were processed within 2 hours.

#### 2.2.2 Instruments and laboratory analysis

Beckman Coulter AU5800 clinical chemical analyzer (Beckman Coulter, USA), Mindray BC-6800 Plus automatic hematology analyzer (Mindray, China), and Alifax erythrocyte sedimentation rate analyzer (ALIFAX, Italy) were used for blood tests. Inova QUANTA-Lyser 160 EIA/IFA Processor (Inova, USA) was used to conduct indirect immunofluorescence assay for manual observation of HEp-2 cells under a microscope, and enzyme-linked immunosorbent assay (ELISA) for anti-dsDNA antibody detection. Assays were performed according to the manufacturer’s instructions, with adherence to standard operation procedures and quality control measured for parameter accuracy. The laboratory indicators measured included creatinine (CREA), blood urea nitrogen (BUN), complement 3 (C3), complement 4 (C4), erythrocyte sedimentation rate (ESR), C-reactive protein (CRP), Lupus anticoagulant (LAC), high-density lipoprotein cholesterol (HDL-C), low-density lipoprotein cholesterol (LDL-C), total cholesterol (TC), and triglyceride (TG).

#### 2.2.3 Disease activity assessment

The disease activity of SLE patients was assessed using SLEDAI-2K, an internationally applied tool. Patients were divided into two groups according to their SLEDAI-2K scores: mild disease activity group (SLEDAI-2K < 10 points) and moderate to severe disease activity group (SLEDAI-2K ≥ 10 points) (16). The SLEDAI-2K scoring system consists of 24 weighted descriptors, and the patient’s total score ranges from 0 to 105 points.

### 2.3 Calculations of systemic inflammation-related indices


$$\begin{array}{l} \text{SII}\;=\;\text{neutrophils}\times \text{platelets}/\text{lymphocyts}\\ \text{SIRI}\;=\;\text{neutrophils}\times \text{monocytes}/\text{lymphocytes}\\ \text{Neutrophil}-\text{to}-\text{lymphocyte}\;\text{ratio}\;\left(\text{NLR}\right)\;=\;\text{neutrophils}/\text{lymphocytes}\\ \text{Platelet}-\text{to}-\text{lymphocyte}\;\text{ratio}\;\left(\text{PLR}\right)\;=\;\text{platelets}/\text{lymphocytes}\\ \text{Monocyte}-\text{to}-\text{lymphocyte}\;\text{ratio}\;\left(\text{MLR}\right)\;=\;\text{monocytes}/\text{lymphocytes} \end{array}$$

### 2.4 Statistical analysis

Graphpad Prism9 software was used for statistical analysis. For continuous data, the Chen-Shapiro test was used to verify normality analysis. Normally distributed data were presented as mean and standard deviation (mean ± SD), with Student’s *t*-test used for comparison between two groups. Non-normally distributed data were presented as median (M) [interquartile range (*P_25_-P_75_*)], with the Mann-Whitney *U*-test for comparison between two groups. Categorical data were presented as percentages (%), and the Chi-squared test was used for pairwise comparison. Pearson’s correlation analysis was used to evaluate the correlation between SIRI and SII with CREA, BUN, C3, C4, ESR, CRP, LAC, HDL-C, LDL-C, TC, TG and SLEDAI-2K. Logistic regression analysis was used to assess the risk factors of SIRI and SII in SLE with LN. Three logistic regression models were constructed: unadjusted model, adjusted model 1 (adjusted for age, gender and disease activity) and adjusted model 2 (further adjusted for CREA, BUN, C3, C4, ESR, CRP, LAC, HDL-C, LDL-C, TC, and TG). Multivariate logistic regression was used to analyze the independent effects of SIRI and SII on SLE. Receiver operating characteristic (ROC) curve was used to evaluate the diagnostic value of SIRI and SII in SLE. A value of p<0.05 was considered statistically significant.

## 3 Results

### 3.1 Demographic data and laboratory findings of patients and controls

**Figure 1:**
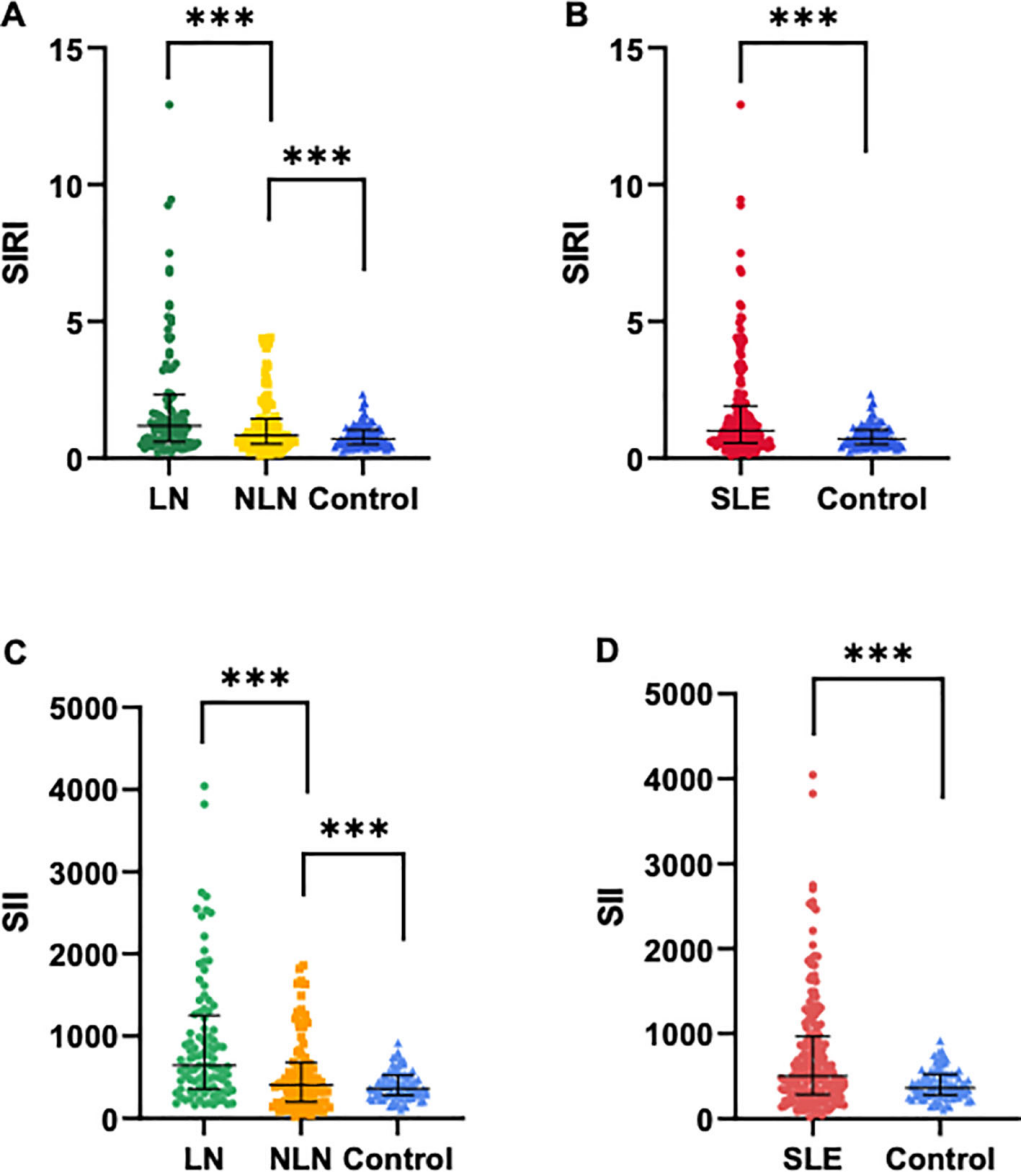
Comparisons of SIRI and SII levels among different groups. **(A, C)** SIRI and SII levels in SLE patients with LN and without LN, and healthy controls. **(B, D)** SIRI and SII levels in SLE patients (N=189) and healthy controls (N=91). Data are expressed as median and interquartile range. *** p<0.001.

The demographic and laboratory indicators of all subjects are shown in **Table 1**. No significant differences were observed in gender and age among the groups. However, there were statistically significant differences in ESR, CREA, BUN, C3, NLR, MLR, PLR, SIRI and SII among the three groups (p<0.05). Additionally, the median levels of SIRI and SII in the SLE group were significantly higher than those in the control group (SIRI: 1.00 vs. 0.71, SII: 502.9 vs. 362.1, p<0.001). Similarly, the median levels of SIRI and SII were significantly higher in the LN group compared to the NLN group and the control group (p<0.001), as shown in **Figure 1**.

**Table 1:** Demographic, clinical, and laboratory characteristics among different groups.

		LN Group	NLN Group	Control Group	t/F/χ^2^	p-Value
No. of participants, N (%)		96 (34.3%)	93 (33.2%)	91 (32.5%)		
Gender	Male, N (%)	17 (17.7%)	9 (9.7%)	16 (17.6%)	3.10	0.213
	Female, N (%)	79 (82.3%)	84 (90.3%)	75 (82.4%)		
Age (years), mean ± SD		40.58 ± 14.99	41.89 ± 17.08	40.40 ± 8.57	0.31	0.732
Lupus nephritis class [N (%)]						
Class I		2 (2.1%)				
Class II		3 (3.1%)				
Pure class III		15 (15.6%)				
Pure class IV		12 (12.5%)				
Pure class V		29 (30.2%)				
Mixed class V		35 (36.5%)				
Laboratory results median (25% Percentile, 75% Percentile)						
ESR (mm/h)		29.00 (16.00, 64.00)	20.00 (9.00, 54.00)	5.00 (4.00, 8.00)	45.60	<0.001
CREA (µmol/L)		77.50 (62.50, 123.00)	55.50 (49.00, 67.75)	65.00 (49.00, 73.00)	21.08	<0.001
BUN (mmol/L)		7.05 (4.53, 12.08)	4.70 (3.88, 5.85)	5.30 (4.60, 6.20)	22.90	<0.001
CRP (mg/L)		4.00 (1.14, 8.00)	3.00 (1.00, 7.00)	4.00 (3.00, 5.00)	2.72	0.067
LAC		1.08 (1.05, 1.18)	1.09 (1.05, 1.21)	--	0.63	0.530
C3 (g/L)		0.57 (0.36, 0.77)	0.65 (0.46, 0.94)	--	2.24	0.026
C4 (g/L)		0.10 (0.05, 0.20)	0.11 (0.05, 0.19)	--	0.16	0.872
TC (mmol/L)		4.63 (3.59, 5.28)	4.11 (3.08, 4.87)	4.35 (3.60, 5.12)	1.44	0.239
TG (mmol/L)		1.70 (1.28, 2.02)	1.31 (1.03, 1.83)	1.20 (0.91, 1.68)	0.82	0.440
LDL-C (mmol/L)		2.74 (2.04, 3.34)	2.25 (1.51, 3.09)	2.63 (2.05, 3.29)	2.13	0.115
HDL-C (mmol/L)		1.01 (0.83, 1.24)	1.02 (0.76, 1.35)	1.03 (0.87, 1.23)	0.06	0.938
NLR		3.31 (2.41, 5.00)	2.81 (1.72, 4.59)	2.14 (1.56, 2.66)	14.97	<0.001
PLR		200.4 (134.7, 259.9)	136.4 (83.33, 212.8)	101.9 (83.43, 124.3)	17.92	<0.001
MLR		0.34 (0.23, 0.53)	0.34 (0.21, 0.45)	0.19 (0.17, 0.25)	30.90	<0.001
SIRI		1.19 (0.59, 2.32)	0.83 (0.52, 1.45)	0.71 (0.50, 1.03)	15.79	<0.001
SII		643.9 (357.0, 1254.0)	406.5 (199.3, 680.3)	362.1 (280.3, 523.3)	24.09	<0.001

ESR, Erythrocyte Sedimentation Rate; CREA, Creatinine; BUN, Blood Urea Nitrogen; CRP, C-Reactive Protein; LAC, Lupus Anticoagulant; C3, Complement 3; C4, Complement 4; TC, Total Cholesterol; TG, Triglycerides; LDL-C, Low-Density Lipoprotein Cholesterol; HDL-C, High-Density Lipoprotein Cholesterol; NLR, Neutrophil-to-Lymphocyte Ratio; PLR, Platelet-to-Lymphocyte Ratio; MLR, Monocyte-to-Lymphocyte Ratio; SIRI, Systemic Inflammation response Index; SII, Systemic Immune-Inflammation Index.

### 3.2 Logistic regression analysis of laboratory indicators in predicting LN risk

Univariate logistic regression analysis showed that SIRI and SII were risk factors for LN (OR_SIRI_: 1.321, 95% CI: 1.081 - 1.614, OR_SII_: 1.002, 95% CI: 1.001 - 1.004). Multivariate regression analysis showed that SIRI and SII were independent risk factors for the occurrence of LN in SLE patients (OR_SIRI_: 5.363, 95% CI: 1.931 - 14.893, OR_SII_: 1.005, 95% CI: 1.002 - 1.007), as shown in **Supplementary Table S1**. To further analyze the predictive value of SIRI and SII for LN, we divided SIRI and SII into quartiles (*P_25_ ~ P_75_*) based on the median (M) and performed multivariable logistic regression. After adjusting for confounding factors (age, gender, disease activity, ESR, LAC, CREA, BUN, C3, C4, TC, TG, HDL-C, LDL-C, and CRP), SIRI (0.56 < SIRI ≤ 1.00 and SIRI > 1.90) was independently associated with LN, with SIRI ≤ 0.56 as the reference group group(Q1). Similarly, SII (294.5 < SII ≤ 502.9) was independently associated with LN, with SII ≤ 294.5 as the reference group(Q1), as shown in **Table 2**.

**Table 2:** Individual effect of SIRI and SII on LN.

	Non-adjusted		Adjusted model I		Adjusted model II	
	OR (95% CI)	p-Value	OR (95% CI)	p-Value	OR (95% CI)	p-Value
SIRI groups						
Q1 (SIRI ≤ 0.56)	Reference		Reference		Reference	
Q2 (0.56 < SIRI ≤ 1.00)	1.13 (0.50, 2.58)	0.77	0.61 (0.17, 2.17)	0.44	0.07 (0.01, 0.16)	0.01
Q3 (1.00 < SIRI ≤ 1.90)	2.25 (0.99, 5.11)	0.05	1.13 (0.35, 3.63)	0.84	0.06 (0.01, 4.02)	0.19
Q4 (SIRI > 1.90)	2.06 (0.91, 4.67)	0.08	0.81 (0.20, 3.22)	0.76	0.05 (0.01, 0.14)	0.01
SII groups						
Q1 (SII ≤ 294.5)	Reference		Reference		Reference	
Q2 (294.5 < SII ≤ 502.9)	1.90 (0.81, 4.45)	0.14	5.39 (1.32, 22.03)	0.02	7.52 (2.94, 19.26)	0.02
Q3 (502.9 < SII ≤ 946.7)	4.16 (1.75, 9.86)	<0.01	7.33 (1.76, 30.48)	<0.01	3.00 (0.77, 14.16)	0.07
Q4 (SII > 946.7)	4.30 (1.82, 10.16)	<0.01	5.75 (1.43, 23.18)	0.01	4.37 (0.41, 46.22)	0.11

Adjusted Model I: Adjusted for age, gender and disease activity.

Adjusted Model II: Further adjusted for ESR, LAC, CREA, BUN, C3, C4, TC, TG, HDL-C, LDL-C, and CRP.

### 3.3 Correlation between SIRI and SII with ESR, LAC, CREA, BUN, C3, C4, TC, TG, HDL-C, LDL-C, CRP and SLEDAI-2K in the SLE group

**Figure 2:**
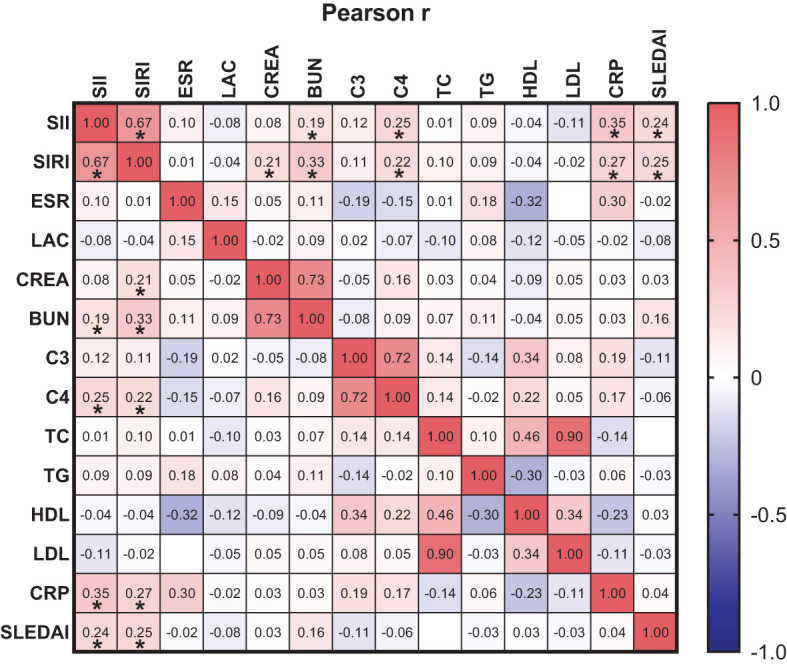
orrelation between SIRI and SII with ESR, LAC, CREA, BUN, C3, C4, TC, TG, HDL-C, LDL-C, CRP and SLEDAI-2K. * p<0.05.

Pearson linear correlation analysis showed that SIRI was positively correlated with CREA, BUN, C4, CRP, and SLEDAI-2K, with correlation coefficients of 0.21, 0.33, 0.22, 0.27, and 0.25, respectively. SII was positively correlated with BUN, C4, CRP, and SLEDAI-2K, with correlation coefficients of 0.19, 0.25, 0.35, and 0.24, respectively. All differences were statistically significant (p<0.05), as shown in **Figure 2**.

### 3.4 ROC curves of SIRI and SII for predicting SLE, SLE disease activity, and LN

**Figure 3:**
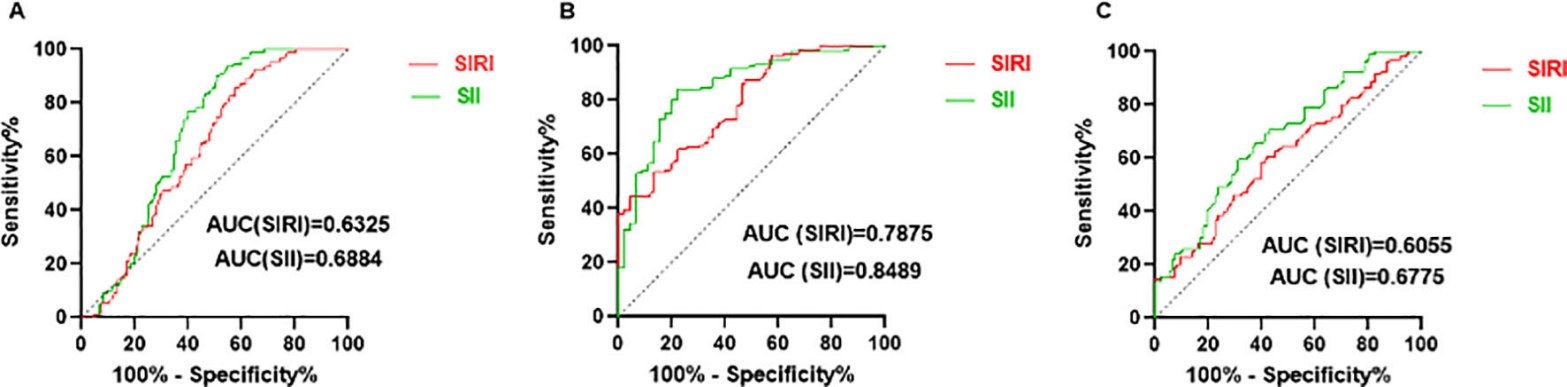
Predicting value of SIRI and SII. **(A)** ROC curves of SIRI and SII for predicting SLE. **(B)** ROC curves of SIRI and SII for predicting moderate to severe SLE activity. **(C)** ROC curves of SIRI and SII for predicting LN.

For predicting SLE, the cut-off values of SIRI and SII were 0.87 (sensitivity: 60.44%, specificity: 60.32%) and 420.3 (sensitivity: 70.33%, specificity: 62.43%), respectively (**Figure 3A**). For predicting mild and moderate to severe activity levels of SLE, the cut-off values of SIRI and SII were 0.79 (sensitivity: 65.97%, specificity: 66.67%) and 359.0 (sensitivity: 79.86%, specificity: 80.00%), respectively (**Figure 3B**).

For predicting LN, SII had the largest area under the ROC curve (AUC) (AUC: 0.6775, 95% CI: 0.6020 - 0.7531). The cut-off values of SIRI and SII were 1.02 (sensitivity: 60.42%, specificity: 60.22%) and 545.9 (sensitivity: 61.46%, specificity: 65.59%), respectively (**Figure 3C**). Results of rest indicators such as NLR (0.5982, 95% CI: 0.5167 - 0.6797), MLR (0.5596, 95% CI: 0.4776 - 0.6417), PLR (0.6567, 95% CI: 0.5779 - 0.7355) are shown in **Supplementary Table S2**.

### 3.5 Comparison of SIRI and SII values across different pathological classes of LN

**Figure 4:**
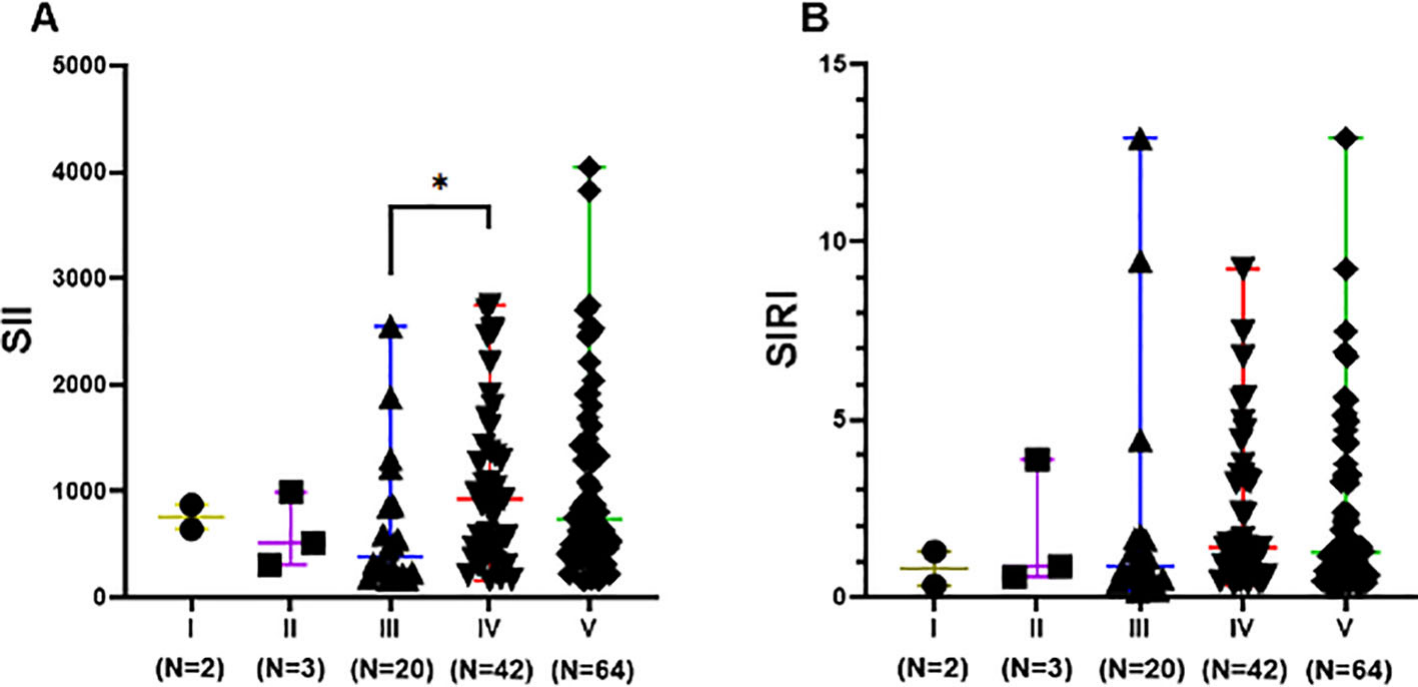
SII and SIRI levels of different pathological classifications in LN group. Data are expressed as median and range. Each dot represents the SII **(A)** and SIRI **(B)** value of each patient. Class III included pure Class III and Class V+III. Class IV included pure Class IV and Class V+IV. Class V included pure Class V, Class V+III, and Class V+IV. * p<0.05.

The difference in SII levels between Class III (including pure Class III and Class V+III) and Class IV (including pure Class IV and Class V+IV) was statistically significant (p<0.05). SII levels in Class III ranged from 169.1 to 2553 with a lower median of 388.3, compared to that in Class IV, which ranged from 160.9 to 2784 with a median of 925.9. However, differences in SIRI levels among different LN pathological classes were not statistically significant (p>0.05), as shown in **Figure 4**.

## 4 Discussion

In patients with SLE, extensive inflammatory responses can lead to multi-organ system damage (17, 18), affecting the central nervous system, hematologic system, skin, musculoskeletal system, lungs, and kidneys. Among these, kidneys are the most affected, with approximately 40%-60% of SLE patients have renal dysfunction (2, 19). LN typically occurs within the first five years of SLE diagnosis (20–22). Previous studies have demonstrated that systemic inflammation in SLE is characterized by elevated markers such as the NLR and PLR, both of which have predictive value (AUC_NLR_: 0.715, AUC_PLR_: less than 0.7, predicting SLE) (11, 23–26). Han et al. (27) reported significantly higher NLR levels in LN patients compared to those without LN. Compared to established markers like NLR and PLR, the SII and SIRI have been less studied, especially in SLE and LN. Our study showed that SII had the largest AUC (AUC: 0.6775, 95% CI: 0.6020 - 0.7531) for predicting LN, higher than NLR (AUC: 0.5982, 95% CI: 0.5167 - 0.6797) and PLR (AUC: 0.6567, 95% CI: 0.5779 - 0.7355). However, the area under the ROC curve for SII was relatively low, so multiple biomarkers should be combined to improve diagnostic and predictive accuracy and clinical utility. Unlike the previous findings by Ozdemir A et al. (28) (NLR was a better marker than SII in predicting SLE and LN), and Gambichler T et al. (29) (SII was significantly increased in SLE patients but was not useful in managing SLE clinically), the reasons for the discrepancies may be due to sample differences (age, race, disease severity) and methodological differences (diagnostic tools, data collection and statistical analysis). These novel inflammatory markers are advantageous due to their simplicity, non-invasiveness, and high sensitivity and specificity, making them effective in identifying kidney damage.

SLE is a complex, multifactorial disease with an unclear etiology. Several factors (genetic, immunologic, endocrine, and environmental) can influence the responses and functions of T and B lymphocytes, as well as innate immune cells via different pathways. Abnormal responses of T cells to autoantigens can lead to cytokine imbalances, including decreased IL-2 and increased IL-17, resulting in tissue inflammatory responses (30, 31). B cells, activated via cytokine interactions with T cells, produce autoantibodies that can infiltrate tissues directly or as immune complexes (IC). This process triggers complement activation, neutrophil activation, and cytokine production, all of which contribute to tissue inflammatory damage. Furthermore, innate immune cells, influenced by pathogenic factors, produce cytokines (such as interferon-α) or interact directly with lymphocytes, significantly promoting tissue inflammatory damage (32). Therefore, inflammation is a critical element in the pathogenesis of SLE. Clinically, SIRI and SII provide comprehensive insights into the inflammatory status of patients by combining multiple blood cell counts, which allows for a more subtle assessment compared to single markers like CRP and ESR. Our study revealed that SII and SIRI values in SLE patients (N = 189) were greater than those in healthy control group (N=91). The SII and SIRI levels in the LN group were higher than those in the non-LN group and healthy control group (all p< 0.001). The ROC curve analysis showed that SII and SIRI are reliable predictors of disease activity and LN. Logistic regression analysis further identified that SII and SIRI are risk factors for LN (OR_SIRI_: 5.363, 95% CI: 1.931 - 14.893, OR_SII_: 1.005, 95% CI: 1.002 - 1.007), with higher SII and SIRI levels correlating with increased LN risk. Compared to other indicators such as CRP, ESR, C3, and C4, SII and SIRI have greater predictive value. Moreover, after controlling for confounding factors, SIRI (0.56 < SIRI ≤ 1.00 and SIRI > 1.90) and SII (294.5 < SII ≤ 502.9) remained independent predictors of LN.

CREA and BUN rise as renal function declines in SLE patients, which is consistent with our study. Accelerated ESR indicates increased RBC aggregation, seen in vascular inflammation and tissue necrosis, and may increase in SLE patients (33). Dyslipidemia, with elevated TC, TG, LDL-C and decreased HDL-C, is associated to autoimmune diseases like SLE and rheumatoid arthritis, promoting inflammation and autoimmunity (34, 35). In SLE patients, dyslipidemia mainly shows as elevated TG, LDL-C, and decreased HDL-C (36). The complement system, with over 30 proteins, involves activation pathways related to SLE inflammation and tissue damage, especially the classical pathway (37). Low C3 and C4 levels are useful diagnostic markers for SLE (38). In this study, we found that SII and SIRI had a non-significant positive correlation with C3. The possible reason for this is that during periods of inflammation, the body's immune system is activated, and the complement system, as a part of it, plays a regulatory and enhancing role in the immune response. When inflammatory markers are elevated, they are often accompanied by increased levels of complement proteins such as C3. The SLEDAI-2K is a weighted index used to evaluate disease activity in SLE (16). Higher SLEDAI-2K scores indicate more affected tissues and organs. Patients with SLE-LN and high disease activity often experience renal involvement, suggesting that higher SLEDAI-2K scores are associated with an increased risk of renal involvement. Our study observed a trend of rising SII and SIRI with increasing SLEDAI-2K scores (rSII=0.23, p<0.05; rSIRI=0.22, p<0.05), consistent with the findings of Ergun, M C (39) (r=0.186; p<0.05), indicating a strong correlation between SII, SIRI, and SLE disease activity. These suggest that SII and SIRI may be more reflective of disease activity in SLE patients.

SII and SIRI are recently identified inflammatory markers mainly used for predicting the development and prognosis of tumors (40–43). These markers also play a significant role in predicting the progression of inflammatory diseases such as cardiovascular diseases and chronic obstructive pulmonary disease (COPD) (44–47). However, their application in SLE and LN is rarely studied. We demonstrated that elevated SII and SIRI values were associated with more severe systemic inflammatory responses and a higher likelihood of developing SLE, with SII showing a significant predictive value. Additionally, the median SII levels were lower in Class III LN pathology compared to Class IV, suggesting potential clinical significance in the stratification of LN risk.

This study has certain limitations. First, as a small-sample, single-center retrospective study without long-term clinical observation, we cannot establish the causal relationship between SII, SIRI, and disease activity or LN. To fully understand the predictive and prognostic value of SII and SIRI in SLE and LN, prospective studies with larger samples and long-term follow-up are necessary. In addition, our study population consisted of hospitalized SLE patients, which might represent more severe cases compared to outpatients. We excluded patients with incomplete SLE disease activity assessments, which might cause discrepancies. Larger, multi-center, prospective studies are needed to evaluate the clinical application value of SII and SIRI.

In conclusion, SII and SIRI levels are closely related to disease activity and LN in SLE patients. SII and SIRI may serve as predictive factors for LN diagnosis and as potential biological indicators for assessing disease progression. Additionally, the simplicity, cost-effectiveness, and non-invasiveness make them easy to popularize and important for early diagnosis and disease activity assessment in SLE and LN.

## Data Availability

The original contributions presented in the study are included in the article/**Supplementary Material**. Further inquiries can be directed to the corresponding author.
