# Supplementary material for: SIRI and SII as potential biomarkers of disease activity and lupus nephritis in systemic lupus erythematosus
Source: Front Immunol. 2025 Jan 31;16:1530534. doi: 10.3389/fimmu.2025.1530534 (PMC11825474; doi:10.3389/fimmu.2025.1530534)
Supplement: Supplementary file 1 [file Table1.docx]

**Supplementary Table**

**Table S1. Univariate and Multivariate analysis of laboratory indicators and demographic characteristics of the study participants**

|  | **Univariate logistic regression analysis** | | | **Multivariate logistic regression analysis** | | |
| --- | --- | --- | --- | --- | --- | --- |
| **Indicators** | **OR** | **95% CI** | **p-Value** | **OR** | **95% CI** | **p-Value** |
| SIRI | 1.321 | 1.081 - 1.614 | <0.001 | 5.363 | 1.931 - 14.893 | <0.001 |
| SII | 1.002 | 1.001 - 1.004 | <0.001 | 1.005 | 1.002 - 1.007 | <0.001 |
| ESR (mm/h) | 1.009 | 0.999 - 1.018 | 0.065 | 0.997 | 0.981 - 1.014 | 0.751 |
| CREA (µmol/L) | 1.046 | 1.027 - 1.066 | <0.001 | 1.054 | 1.024 - 1.085 | <0.001 |
| BUN (mmol/L) | 1.243 | 1.117 - 1.383 | <0.001 | 0.968 | 0.793 - 1.183 | 0.754 |
| CRP (mg/L) | 1.005 | 0.987 - 1.024 | 0.606 | 1.004 | 0.974 - 1.035 | 0.774 |
| LAC | 1.362 | 0.518 - 3.584 | 0.531 | 0.763 | 0.219 - 2.660 | 0.671 |
| C3 (g/L) | 0.326 | 0.120 - 0.884 | 0.028 | 0.954 | 0.102 - 8.921 | 0.967 |
| C4 (g/L) | 0.630 | 0.036 - 11.002 | 0.751 | 0.003 | 0.001 - 2.765 | 0.096 |
| TG (mmol/L) | 1.334 | 0.900 - 1.977 | 0.151 | 1.526 | 0.831 - 2.799 | 0.173 |
| TC (mmol/L) | 1.206 | 0.954 - 1.525 | 0.117 | 0.755 | 0.232 - 2.458 | 0.640 |
| HDL-C (mmol/L) | 0.998 | 0.454 - 2.194 | 0.995 | 2.609 | 0.422 - 16.127 | 0.302 |
| LDL-C (mmol/L) | 1.299 | 0.970 - 1.739 | 0.079 | 2.214 | 0.582 - 8.426 | 0.244 |
| NLR | 1.078 | 0.994 - 1.168 | 0.061 | 1.031 | 0.892 - 1.190 | 0.682 |
| MLR | 4.363 | 1.055 - 18.039 | 0.042 | 1.142 | 0.076 - 17.078 | 0.932 |
| PLR | 1.001 | 0.999 - 1.003 | 0.149 | 1.002 | 0.999 - 1.006 | 0.170 |

ESR: Erythrocyte Sedimentation Rate, CREA: Creatinine, BUN: Blood Urea Nitrogen, CRP: C-Reactive Protein, LAC: Lupus Anticoagulant, C3: Complement 3, C4: Complement 4, TC: Total Cholesterol, TG: Triglycerides, LDL-C: Low-Density Lipoprotein Cholesterol, HDL-C: High-Density Lipoprotein Cholesterol, NLR: Neutrophil-to-Lymphocyte Ratio, PLR: Platelet-to-Lymphocyte Ratio, MLR: Monocyte-to-Lymphocyte Ratio, SIRI: Systemic inflammation response index, SII: Systemic Immune-Inflammation Index

**Table S2. Sensitivity, specificity, cut-off value, and ROC curve analysis results of predicting LN for each indicator**

| **Indicators** | **AUC** | **Sensitivity** | **Specificity** | **Cut-off Value** | **95%CI** |
| --- | --- | --- | --- | --- | --- |
| SIRI | 0.6055^*^ | 60.42% | 60.22% | 1.02 | 0.5254 - 0.6856 |
| SII | 0.6775^*^ | 61.46% | 65.59% | 545.9 | 0.6020 - 0.7531 |
| CRP (mg/L) | 0.5203 | 54.12% | 52.33% | 3.18 | 0.4334 - 0.6072 |
| NLR | 0.5982^*^ | 59.38% | 53.76% | 2.91 | 0.5167 - 0.6797 |
| MLR | 0.5596 | 51.04% | 49.46% | 0.34 | 0.4776 - 0.6417 |
| PLR | 0.6567^*^ | 60.42% | 63.44% | 178.7 | 0.5779 - 0.7355 |
| WBC (10^9^/L) | 0.5591 | 55.21% | 53.76% | 4.92 | 0.4771 - 0.6412 |
| NE (10^9^/L) | 0.5904^*^ | 56.25% | 55.91% | 3.20 | 0.5095 - 0.6712 |
| LY (10^9^/L) | 0.5438 | 53.13% | 52.69% | 1.06 | 0.4603 - 0.6273 |
| MO (10^9^/L) | 0.5248 | 52.08% | 53.76% | 0.34 | 0.4457 - 0.6112 |

* p<0.05

NLR: Neutrophil-to-Lymphocyte Ratio, PLR: Platelet-to-Lymphocyte Ratio, MLR: Monocyte-to-Lymphocyte Ratio, SIRI: Systemic inflammation response index, SII: Systemic Immune-Inflammation Index, NE: Neutrophil, LY: Lymphocyte, MO: Monocyte
